# Neutrophils Release Metalloproteinases during Adhesion in the Presence of Insulin, but Cathepsin G in the Presence of Glucagon

**DOI:** 10.1155/2018/1574928

**Published:** 2018-02-14

**Authors:** Natalia V. Fedorova, Alexander L. Ksenofontov, Marina V. Serebryakova, Vladimir I. Stadnichuk, Tatjana V. Gaponova, Ludmila A. Baratova, Galina F. Sud'ina, Svetlana I. Galkina

**Affiliations:** ^1^A. N. Belozersky Institute of Physico-Chemical Biology, Lomonosov Moscow State University, Moscow 119991, Russia; ^2^Physical Department, Lomonosov Moscow State University, Moscow 119991, Russia; ^3^FGBU Hematology Research Center, Russian Federation Ministry of Public Health, Moscow 125167, Russia

## Abstract

In patients with reperfusion after ischemia and early development of diabetes, neutrophils can attach to blood vessel walls and release their aggressive bactericide agents, which damage the vascular walls. Insulin and 17*β*-estradiol (E2) relieve the vascular complications observed in metabolic disorders. In contrast, glucagon plays an essential role in the pathophysiology of diabetes. We studied the effect of hormones on neutrophil secretion during adhesion to fibronectin. Amino acid analysis revealed that proteins secreted by neutrophils are characterized by a stable amino acid profile enriched with glutamate, leucine, lysine, and arginine. The total amount of secreted proteins defined as the sum of detected amino acids was increased in the presence of insulin and reduced in the presence of glucagon. E2 did not affect the amount of protein secretion. Proteome analysis showed that in the presence of insulin and E2, neutrophils secreted metalloproteinases MMP-9 and MMP-8 playing a key role in modulation of the extracellular matrix. In contrast, glucagon induced the secretion of cathepsin G, a key bactericide protease of neutrophils. Cathepsin G can promote the development of vascular complications because of its proinflammatory activity and ability to stimulate neutrophil adhesion via the proteolysis of surface receptors.

## 1. Introduction

Neutrophils, or polymorphonuclear leukocytes, play an important role in host defense against bacterial or fungal pathogens due to their ability to penetrate into infected tissue and phagocytose and kill microbes. To kill microbes, bactericide agents that are localized in the neutrophil secretory granules are released into the formed phagosomes and outwards [1]. Bactericides, being released outside, cause the destruction of surrounding host tissues and the development of inflammation.

Neutrophils can release their bactericides to the outside also in the absence of infection. In patients with certain metabolic disorders, neutrophils that normally roll in the bloodstream can attach to the walls of the blood vessels and secrete their aggressive products. Adherence of neutrophils to endothelial cells is an early and requisite event in ischemia/reperfusion-induced inflammatory injury [2, 3]. The authors of many modern studies consider the integrin-dependent adhesion of neutrophils and associated secretion of neutrophil bactericidal agents to be the cause of the early stages of retinopathy [4–6] or nephropathy [7, 8] in patients with diabetes.

Insulin produced by *β*-cells of the pancreatic Langerhans islets plays a key role in maintaining vascular health. Clinical evidence indicates that intensive insulin therapy protects the endothelium in cardiovascular diseases, during critical illness [9, 10], and reduces major cardiovascular events in diabetics [11]. In particular, insulin attenuates neutrophil accumulation in myocardial ischemia/reperfusion rabbit hearts and inhibits neutrophil adhesion to cultured endothelial cells subjected to simulated ischemia/reperfusion [12].

Glucagon produced by *α*-cells of the Langerhans islets is a physiological antagonist of insulin. In healthy patients, production of glucagon is inhibited by insulin at a high level of glucose in the blood. In patients with diabetes, an oral glucose load induced a paradoxical rise in glucagon secretion. Absolute or relative hyperglucagonaemia has been recognized for years in all experimental or clinical forms of diabetes [13]. Glucagon seems to play an essential role in the pathophysiology of diabetes. Knockout of the glucagon receptor or administration of a monoclonal specific glucagon receptor antibody makes insulin-deficient type 1 diabetic rodents thrive without insulin [14–16]. However, the mode of glucagon action remains to be elucidated. Despite the active search, a glucagon antagonist, which can be effective in treating diabetes, has not yet been found. In the treatment of type 2 diabetes, there are largely used drugs that act, in part, by inhibiting the secretion or action of glucagon, such as glucagon-like peptide-1 (GLP-1) and GLP-1 receptor agonists, dipeptidyl peptidase-4 inhibitors (DPP-4), and metformin [17–19]. Inhibition of glucagon secretion and stimulation of insulin release equally contribute to a decrease in glucose with respect to the action of GLP-1 [17].

The action of GLP-1 is transient, since the peptide is rapidly inactivated by DPP-4. DPP-4 inhibitors are used to prolong the action of GLP-1. Metformin has a glucose-lowering effect, inhibiting hepatic gluconeogenesis and counteracting the action of glucagon [19]. GLP-1 receptor agonists, DPP-4 inhibitors, and metformin have beneficial effects on cardiovascular complications in patients with type 2 diabetes, as well as in patients with reperfusion after ischemia [20–22]. Prevention of vascular adhesion of monocytes contributes to the cardiovascular protective effect of GLP-1 analogs [23, 24]. Metformin also could reduce vascular complication of diabetes by decreasing leukocyte oxidative stress and undermining adhesion molecule levels and leukocyte-endothelium interactions [25].

Female hormones play an important role in maintaining vascular health, as evidenced by an increase in the incidence of cardiovascular disease in women after menopause [26]. Pretreatment with estrogen prior to myocardial ischemia and reperfusion causes a decrease in neutrophil infiltration into the irreversibly injured myocardium [27]. Traditionally, estrogen acts via classical nuclear estrogen receptors. In addition to this “genomic” signalling pathway, a “rapid, nonnuclear” signalling pathway mediated by cell membrane-associated estrogen receptors also has been recognized. This nonnuclear signalling appears to be critical for the protective effects of estrogen in the cardiovascular system [28–30].

In this work, we studied the effect of insulin, glucagon, and 17*β*-estradiol (E2) on secretion of human neutrophils upon adhesion to fibronectin. We used scanning electron microscopy to study the morphology of the attached neutrophils, as well as amino acid analysis and mass spectrometry to study the amount and composition of secreted proteins.

## 2. Material and Methods

### 2.1. Materials

Ficoll-Paque for neutrophil isolation was obtained from Pharmacia (Uppsala, Sweden). Fibronectin was from Calbiochem (La Jolla, USA). Bicarbonate-free Hank's solution, Ca^+^-free Dulbecco PBS, insulin, glucagon, and 17*β*-estradiol were purchased from Sigma. Trypsin was from Promega, and Coomassie Brilliant Blue G-250 was from Serva.

### 2.2. Neutrophil Isolation

Neutrophils were isolated from the blood of healthy volunteers, who did not take any medication for 2 weeks, using experimental procedures approved by the Ethics Committee of A. N. Belozersky Institute. Erythrocytes were precipitated with 3% dextran T-500 at room temperature. Neutrophils were isolated from the plasma by centrifugation via Ficoll-Paque (density 1.077 g/mL). The remaining red blood cells were eliminated by hypotonic lysis. After washing, neutrophils (purity 96-97%, viability 98-99%) were stored before the experiment in Dulbecco's PBS containing 1 mg/mL glucose (without CaCl_2_).

### 2.3. Adhesion of Neutrophils to Fibronectin-Coated Cover Slips

For scanning electron microscopy (SEM), neutrophils were plated onto fibronectin-coated glass coverslips. The coverslips were coated with fibronectin for 2 hours by incubation in Hank's solution containing 5 *μ*g/mL of fibronectin at room temperature and washed with buffer. Neutrophils adhered to the fibronectin-coated coverslips (3 × 10^6^ cells in 2 mL per well) for a 20 min incubation in Hank's solution containing 10 mM HEPES (pH 7.35) at 37°C. Insulin, glucagon, or 17*β*-estradiol (0.1 *μ*M) was added to the cells before plating. After that, the cells were fixed for SEM.

### 2.4. Sampling of the Extracellular Medium to Determine the Amino Acid and Protein Composition of Neutrophil Secretion

Six-well culture plates were coated with fibronectin for 2 hours of incubation in Hank's solution containing 5 *μ*g/mL fibronectin at room temperature and washed. Neutrophils adhered to the protein-coated wells (3 × 10^6^ cells in 1.3 mL per well) in Hank's solution containing 10 mM HEPES (pH 7.35) for 20 minutes at 37°C. Insulin (0.1 *μ*M), glucagon (0.1 *μ*M), or 17*β*-estradiol (0.1 *μ*M) was added to the cells prior to plating. After the incubation, the extracellular medium was sampled from the control and hormone-treated cells. Aliquots were collected from six similar wells and combined. Inhibitors of metalloproteinase, serine and cysteine proteinases, and myeloperoxidase (EDTA, 5 mM; PMSF, 200 *μ*M; E64, 10 *μ*M; and sodium azide, 0.025%, resp.) were immediately added to the samples. Unattached neutrophils were removed by centrifugation at 2000*g*.

### 2.5. Extraction, Concentration, and Hydrolysis of Proteins

Proteins from the collected EM samples were extracted with an equal volume of a chloroform-methanol mixture (2 : 1, *v*/*v*). The mixture was vortexed for 1-2 minutes and stirred in a shaker at 4°C for 30 minutes. The chloroform and methanol phases were then separated by centrifugation at 11,000*g* for 20 minutes, and the solvents were evaporated. The chloroform phase contained almost all of the proteins detected. The water-methanol fractions contained trace amounts of protein. Concentrated extracts of chloroform phases were subjected to electrophoresis. For amino acid analysis, the same extracts were prehydrolysed, as described by Tsugita and Scheffler [31].

### 2.6. Quantitative Determination of Amino Acids with Ninhydrin

The amino acid profile of protein hydrolysates was determined by an L-8800 amino acid analyser (Hitachi, Tokyo, Japan) with an electronic heating bath and two single-channel colorimeters according to the manufacturer's user manual (Hitachi High-Technologies Corporation, Japan, 1998). The protein hydrolysates were separated on a 2622SC-PF ion-exchange column (Hitachi Ltd., P/N 855-4507, 4, 6 × 60 mm) by a step gradient of four Li-citrate buffers at a flow rate of 0.35 mL/min and a thermostating column at 30–70°C. Postcolumn derivatization (136°C, flow rate 0.35 mL/min) was performed using a mix of equal volumes of ninhydrin buffer R2 and ninhydrin solution R1 (Wako Pure Chemical Industries, P/N 298-69601). Colored products were detected by measuring the absorbance at 570 nm for all amino acids except proline and at 440 nm for proline. Data were processed using MultiChrom for Windows software (Ampersand Ltd., Moscow, Russia).

The total amount of proteins released by control cells was defined as the sum of detected amino acids (Table 1). Insulin and glucagon are protein hormones with a molecular mass of 5800 and 3482, respectively. When used at 0.1 *μ*M, they contribute 0.58 or 0.35 *μ*g of protein per millilitre. Bearing in mind that the total volume of each sample is 7.8 mL (1.3 mL × 6), we subtracted 4.5 or 2.7 *μ*g, respectively, from the total amount of proteins secreted by insulin- or glucagon-treated cells.

### 2.7. Sodium Dodecyl Sulfate Polyacrylamide Gel Electrophoresis

Protein separation was performed using one-dimensional sodium dodecyl sulfate electrophoresis on a 15% polyacrylamide gel under nonreducing conditions in the Mini-PROTEAN 3 Cell (Bio-Rad) [32]. Prior to electrophoresis, aliquots of the preparations were boiled for 3 minutes in lysis buffer (Tris-HCl 30 mM, pH 6.8; SDS 1%; urea 3 M; glycerin 10%; bromophenol blue 0.02%). Gels were stained with Coomassie Brilliant Blue G-250 0.22% (Serva).

### 2.8. Mass Spectrometry Identification of Proteins and Preparation of Samples

A MALDI-time of flight (ToF)-ToF mass spectrometer (Ultraflex II Bruker, Germany) equipped with a neodymium-doped (Nd) laser was used for matrix-assisted laser desorption ionization mass spectrometry (MALDI-MS) and tandem mass spectrometry (MS/MS) analysis of proteins. Proteins separated by electrophoresis were subjected to trypsin hydrolysis directly in the gel. To this end, after electrophoresis, 1 × 1 mm slices of gel were cut from each Coomassie-stained protein band. Gel pieces were washed twice with 100 *μ*L acetonitrile 40% in NH_4_HCO_3_ 100 mM (pH 7.5) for 30 min at 37°C, dehydrated with 100 *μ*L acetonitrile, and air-dried. Then, they were incubated with 4 *μ*L modified trypsin 12 *μ*g/mL (Promega) in NH_4_HCO_3_ 50 mM for 6 h at 37°C. The resulting peptides were recovered through incubation with 6 *μ*L trifluoroacetic acid solution 0.5% in acetonitrile 10% for 30 min. For mass spectrometric analysis, sample aliquots (1 *μ*L) were mixed on a steel target with 0.3 *μ*L 2,5-dihydroxybenzoic acid (20 mg/mL in acetonitrile 20% and trifluoroacetic acid 0.5%) and air-dried at room temperature. The [MH]^+^ molecular ions were measured in reflector mode; the accuracy of mass peak measurement was within 0.005%. Identification of proteins was carried out by a peptide fingerprint search using Mascot software (http://www.matrixscience.com) through the Unipro+ (SwissPro+) mammalian protein database with the indicated accuracy. The search allowed for possible oxidation of methionine by environmental oxygen and modification of cysteine with acrylamide, and where a score was >71, protein matches were considered significant (*p* < 0.05).

### 2.9. Scanning Electron Microscopy Technique

Neutrophils that were attached to fibronectin were fixed in 2.5% glutaraldehyde in Hanks buffer, which did not contain Ca^2+^ or Mg^2+^ ions, but contained inhibitors of metalloproteinases and serine proteases (5 mM EDTA and 0.5 mM PMSF, resp.) and 10 mM HEPES at pH 7.3. The cells were additionally fixed with 1% solution of osmium tetroxide in 0.1 M sodium cacodylate containing 0.1 M sucrose at pH 7.3. The samples were then dehydrated in an acetone series (10–100%) and dried at a critical point with liquid CO_2_ as the transition liquid in the Balzers apparatus. The samples were sputter-coated with gold/palladium and observed at 15 kV using a Camscan S-2 scanning electron microscope.

## 3. Results and Discussion

### 3.1. Effect of Insulin, E2, and Glucagon on the Morphology of Human Neutrophils Attached to а Fibronectin-Coated Substrate

**Figure 1:**
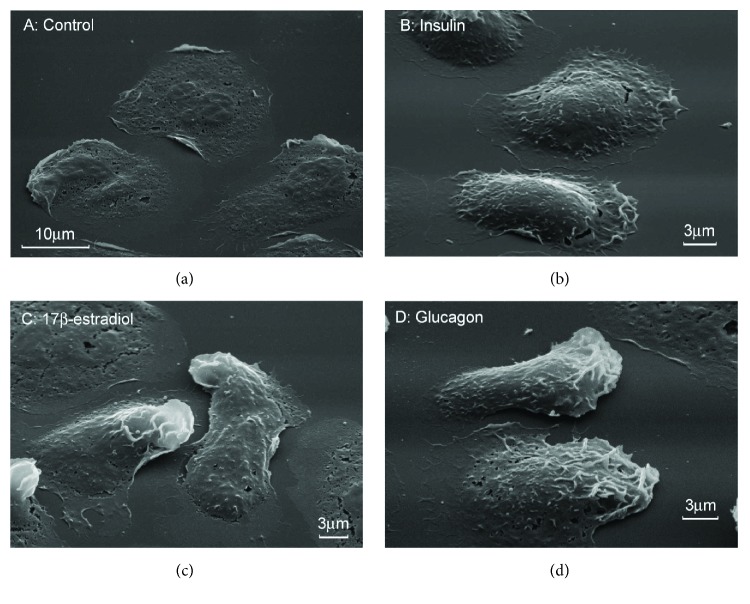
Effect of hormones on the morphology of human neutrophils attached to fibronectin. Scanning electron microscopy images of neutrophils that were attached to fibronectin for 20 min in control conditions (a) or in the presence of 0.1 *μ*M insulin (b), 0.1 *μ*M E2 (c), or 0.1 *μ*M glucagon (d). Pictures represent typical images observed in two independent experiments.

The adhesion of resting neutrophils (control neutrophils) to a glass or polystyrene itself leads to cell activation [33]. We studied the secretion of neutrophils in the process of adhesion to substrates coated with fibronectin, the extracellular matrix protein, since neutrophils exhibit only a priming activation when adhered to fibronectin. We compared the morphology of neutrophils that were attached to fibronectin-coated substrata in the presence 0.1 *μ*M of insulin, glucagon, or E2. Scanning electron microscopy showed that the control cells and insulin-, glucagon-, or E2-treated cells were attached and spread on fibronectin-coated substrates (Figures 1(a)–1(d)). At the concentration used in the experiment, the hormones contribute only minor features to the morphology of the cells.

### 3.2. Effect of Insulin, Glucagon, and E2 on the Amount and Amino Acid Composition of Proteins Released by Neutrophils in Adhesion to Fibronectin

Neutrophils secrete their products via a variety of the mechanisms, including fusion of secretory granules with the plasma membrane [34] and shedding of membrane vesicles (ectosomes) from the plasma membrane [35–39]. Secreted proteins enter the extracellular medium as separate molecules or as part of membrane structures. To analyse all secreted proteins, we extracted proteins from the extracellular medium using a chloroform-methanol mixture. Earlier, we were convinced that practically all proteins are in the chloroform fraction [40]. After evaporation of the solvent, the proteins in the chloroform fraction were separated by electrophoresis or subjected to acid hydrolysis and subsequent amino acid analysis.

**Figure 2:**
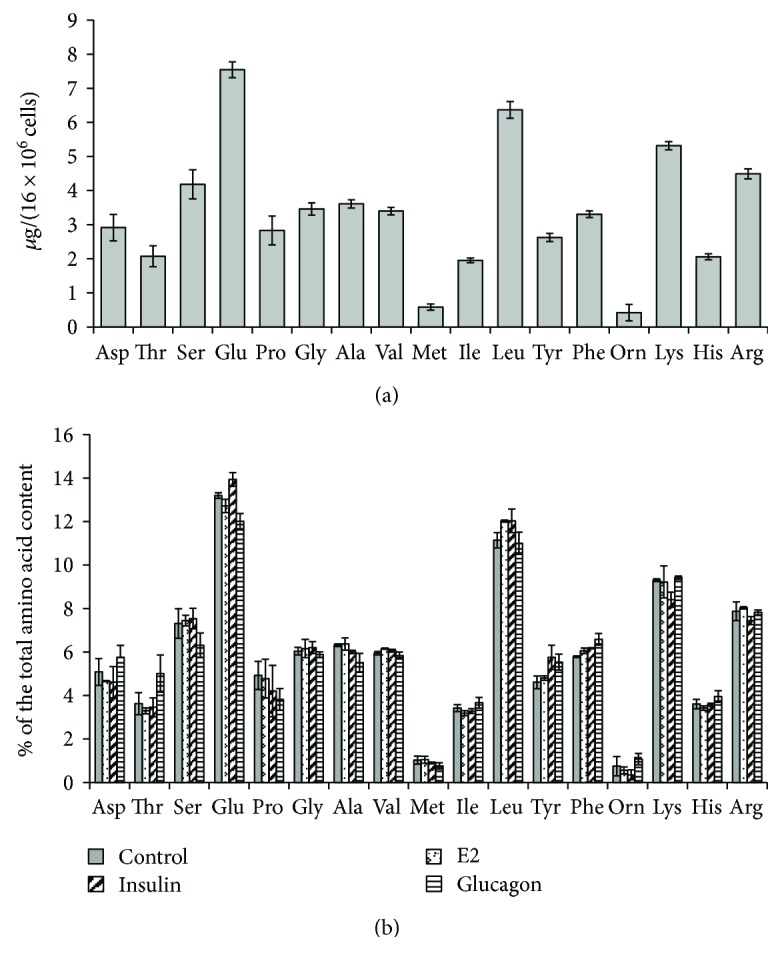
Effect of hormones on the amino acid composition of proteins secreted by neutrophils in adhesion to fibronectin. Human neutrophils were attached to a substrate coated with fibronectin for 20 min under control conditions or in the presence of 0.1 *μ*M E2, insulin, or glucagon. The proteins were extracted from the extracellular medium, concentrated, and after acid hydrolysis subjected to amino acid analysis. (a) The amount of detected amino acids in the hydrolysate of proteins secreted by control neutrophils (mean ± SEM) is presented. (b) Comparison of the percentage of individual amino acids in hydrolysates of proteins secreted by neutrophils under control conditions and in the presence of hormones. Amino acid profiles were obtained by summing the results of three independent experiments.

We used amino acid analysis to determine the total amount of protein secreted by neutrophils during adhesion to fibronectin under various conditions. After acid hydrolysis, the quantitative content of the individual amino acids in the protein hydrolysate was determined using an amino acid analyser (Figure 2).

In the process of acid hydrolysis, tryptophan was destroyed, and glutamine and asparagine were converted into glutamic and aspartic acid, respectively. Therefore, tryptophan is absent from the histogram, and the columns corresponding to glutamic acid and aspartic acid represent the sum of asparagine and aspartic acid or glutamine and glutamic acid, respectively. Amino acid analysis revealed a stable amino acid profile of proteins released by neutrophils that were attached to fibronectin under control conditions. The protein hydrolysate was enriched with serine, glutamate/glutamine, leucine, lysine, and arginine (Figure 2(a)). Summarizing the number of all detected amino acids, we determined the total amount of protein secreted by the cells under different experimental conditions (Table 1). The amount of secreted proteins increased by 18% in the presence of insulin but reduced in the presence of glucagon. The female hormone E2 had little effect on the amount of protein secreted by neutrophils.

A comparison of the percentage of individual amino acids in hydrolysates of proteins showed that the amino acid profile of protein secretion was generally retained for neutrophils that were attached to fibronectin in the presence of insulin, E2, or glucagon (Figure 2(b)). The amino acid profile of secretion products can serve as a type of “fingerprint” of human neutrophil secretion.

### 3.3. Effect of E2 on the Protein Composition of Neutrophil Secretion in Adhesion to Fibronectin

**Figure 3:**
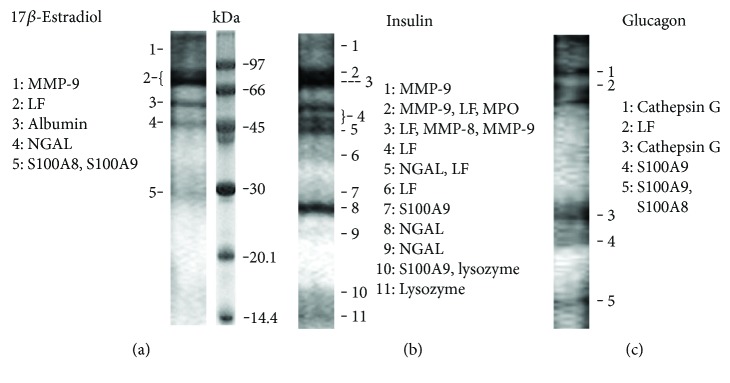
SDS-PAGE separation of proteins secreted by neutrophils upon adhesion to fibronectin in the presence of hormones. Human neutrophils were attached to fibronectin-coated substrata for 20 min in the presence of 0.1 *μ*M E2 (a), 0.1 *μ*M insulin (b), or 0.1 *μ*M glucagon (c). Samples of extracellular medium were collected, and proteins were extracted and subjected to 15% SDS-PAGE. Pictures represent typical protein profiles observed in the three independent experiments for each hormone.

To determine which proteins are secreted by neutrophils under the action of hormones, protein extracts of extracellular media after removal of chloroform were separated by electrophoresis (Figure 3). Identification of proteins represented by separate bands in the gel was carried out by mass spectrometry after trypsin hydrolysis directly in the gel (Table 2).

Previously, we have shown that control neutrophils during 20 min of adhesion to fibronectin secreted lactoferrin (LF), neutrophil gelatinase-associated lipocalin (NGAL), lysozyme, and myeloperoxidase (MPO), as well as albumin and cytosolic S100A8 and S100A9 proteins [40, 41]. Neutrophils contain secretory granules of four types [1]. The composition of granules of different types largely overlaps [42]. Nevertheless, LF and NGAL are considered components of the secondary granules, MPO is contained mainly in the primary granules, and albumin belongs to secretory vesicles.

When neutrophils adhere to fibronectin in the presence of E2, they release the same proteins as the control cells (LF, albumin, NGAL, S100A8, and S100A9) and, in addition, metalloproteinase 9 (MMP-9) (Figure 3(a), Table 2). MMP-9 is localized presumably in the tertiary or gelatinase granules of neutrophils [43]. Metalloproteinases are involved in the adhesion and migration of neutrophils because of their ability to modulate the extracellular matrix and remove the barriers of the basement membranes [44]. The balance between MMPs and their tissue inhibitors (TIMPs) tightly regulates physiological and pathological processes characterized by the degradation and accumulation of the extracellular matrix [45]. We assume that the ability of E2 to initiate the secretion of MMP-9 in adherent neutrophils can alter the result of the interaction of neutrophils with the walls of the blood vessel and contribute to the protective effect of E2 in metabolic diseases.

### 3.4. Effect of Insulin on the Protein Composition of Neutrophil Secretion in Adhesion to Fibronectin

Neutrophils that adhered to fibronectin in the presence of insulin secreted the same proteins as the control cells and, in addition, MMP-9 and matrix metalloproteinase 8 (MMP-8) (Figure 3(b); Table 2). MMP-8, or neutrophil collagenase, is involved in degradation of all structural components of extracellular matrix and plays a crucial role in many physiological processes including inflammatory and cardiovascular disorders [46]. Since many inflammatory processes are accompanied by increased levels of MMP-8, it is assumed that the enzyme contributes to the development of inflammation. However, modern research refutes this view. The emergence of MMP-8-deficient mice resulted in the publication of a number of studies demonstrating an anti-inflammatory role of MMP-8 during lipopolysaccharide- (LPS-) mediated acute lung injury [47, 48] or allergen-induced airway inflammation [49].

Another characteristic feature of insulin-stimulated secretion was the presence of NGAL (neutrophil gelatinase-associated lipocalin) in several electrophoretic bands (Figure 3(b), bands 5, 8, 9) corresponding to monomeric (24 kDa) or homodimeric (46 kDa) forms of the protein [50]. In secretion from control [40] or E2-treated cells (Figure 3(a), band 4), NGAL was represented by minor bands corresponding to its homodimer form. These data indicating that, in the presence of insulin, neutrophils secrete an increased amount of NGAL are consistent with previously published work demonstrating that hyperinsulinaemic induction in humans significantly increases circulating levels of lipocalin-2. Additionally, in omental adipose tissue explants, insulin caused a significant dose-dependent increase in lipocalin-2 protein production and secretion into conditioned media [51].

A characteristic feature of NGAL is the ability to form complexes with MMP-9 after these components are in the extracellular environment. According to some researchers, the complex with NGAL supports allosteric activation of MMP-9 [52]. From another point of view, the formation of complex with NGAL protects MMP-9 from degradation by TIMPs, thereby maintaining the activity of the enzyme [44, 53, 54]. In any case, insulin initiates the secretion of MMP-9 in neutrophils and, at the same time, increases the release of lipocalin, which helps maintain the activity of MMP-9.

### 3.5. Effect of Glucagon on the Protein Composition of Neutrophil Secretion in Adhesion to Fibronectin

The main components of the secretion of neutrophils adherent to fibronectin in the presence of glucagon were the cathepsin G, LF, and S100A9 proteins (Figure 3(c), Table 2). Cathepsin G is one of the three serine proteases (along with proteinase-3 and elastase) that are contained in primary (azurophil) granules of neutrophils and involved in clearance of internalized pathogens, proteolytic modification of chemokines and cytokines, and shedding of cell surface receptors [55]. Cathepsin G deficiency in gene-targeted mice increases susceptibility to *Staphylococcus aureus* and fungal infections indicating the key role of the enzyme in neutrophil antimicrobial activity [56, 57].

The glucagon-induced neutrophil secretion is also enriched in LF. Recent data show that LF can serve as an allosteric enhancer of the proteolytic activity of cathepsin G [58]. LF potently increases the activity of cathepsin G at pH 7.4 and to an even higher extent at pH 5, as well as in granulocyte-derived supernatant. Furthermore, LF might induce a conformational change of cathepsin G resulting in advanced substrate selectivity. LF and cathepsin G appear to act synergistically during secretion by granulocytes augmenting the process associated with host defense. We suggest similar synergistic interactions may occur in blood vessels between cathepsin G and LF that are secreted by glucagon-treated neutrophils attached to the vessel walls in patients with metabolic disorders.

Cathepsin G secreted by neutrophils can damage the vascular walls via promotion of inflammation or disruption of the neutrophil surface receptors. Cathepsin G, for example, is able to cleave leukosialin (CD43), the predominant cell surface sialoprotein of leukocytes, and releases its extracellular domain [59]. The shedding of highly negatively charged membrane sialoglycoprotein CD43 is commonly thought to enhance neutrophil adhesion. Thus, glucagon-induced cathepsin G secretion, in turn, may further potentiate the adhesion of neutrophils and the corresponding damage to blood vessels [60, 61].

### 3.6. Conclusions

Our in vitro experiments revealed that insulin and E2 stimulated secretion of MMP-9 and MMP-8 by human neutrophils during adhesion to fibronectin-covered substrata. In contrast, glucagon stimulated secretion of cathepsin G. We assume that hormones can affect the state of blood vessels in diabetes and metabolic disorders, regulating the adhesion of neutrophils to the walls of blood vessels and their corresponding secretion. Extracellular matrix proteins play a crucial role in the process of cell adhesion. Insulin and E2 can alter the adhesion of neutrophils initiating the secretion of metalloproteinase, which modifies extracellular matrix proteins. Glucagon can contribute to the development of metabolic vascular disorders by initiating the secretion of cathepsin G, the key enzyme of the bactericide activity of neutrophils. Cathepsin G may promote inflammation and stimulate further neutrophil adhesion via proteolysis of cell surface receptors.

## Figures and Tables

**Table 1:** Effect of hormones on the amount of protein secreted by neutrophils in adhesion to fibronectin.

Treatment	Average amount of secreted proteins, *μ*g/18 × 10^6^ cells
Control	64.6 ± 1.8
E2	62.5 ± 3.2
Insulin	76.1 ± 2.0^∗^
Glucagon	50.0 ± 1.9^∗^

Proteins were extracted from extracellular medium samples collected from neutrophils that were attached to fibronectin during a 20 min incubation in the presence of 0.1 *μ*M E2, insulin, or glucagon. After acid hydrolysis, the protein hydrolysates were subjected to amino acid analysis. The amount of secreted proteins was determined as the sum of the amounts of the detected amino acids. ^∗^*p* < 0.05 when compared to the control value.

**Table 2:** List of proteins secreted by neutrophils in adherence to fibronectin in the presence of hormones.

	Protein name				Peptides matched/total	Coverage, %	MOWSE score
	Control	Insulin	E2	Glucagon			
MMP-9_HUMAN		MMP-9	**+**		18/85	25	240
MMP-8_HUMAN		MMP-8			15/85	31	240
CATG_HUMAN				Cath. G	15/77	40	75
PERM_HUMAN	**+**	MPO			17/85	21	119
TRFL_HUMAN	**+**	LF	**+**	+	29/85	43	240
ALBU_HUMAN	+		Albumin		15/28	24	130
NGAL_HUMAN	**+**	NGAL	**+**		7/15	42	105
LYSC_HUMAN	**+**	Lysozyme^∗^	**+**			31	98
S10A9_HUMAN	**+**	S100-A9	**+**	+	9/19	48	147
S10A8_HUMAN	**+**		S100-A8	+	5/30	44	84

Neutrophils were attached to fibronectin for 20 minutes under control conditions (marked +), or in the presence of 0.1 *μ*M insulin, 0.1 *μ*M E2, or 0.1 *μ*g glucagon. Mass spectrometric analysis data were taken from experiments with insulin. Analogous proteins that were identified in control experiments or in experiments with E2 or glucagon are marked (**+**). Mass spectrometric data for cathepsin G are taken from experiments with glucagon, and those for albumin and S100A8 from experiments with E2. Proteins were separated by SDS-PAGE and identified by mass spectrometric analysis. ^∗^Protein was identified by MSMS analysis. Proteins identified in two or three analogous experiments were included in the list.

## Abbreviations

SEM: Scanning electron microscopy
MPO: Myeloperoxidase
Lf: Lactoferrin
NGAL: Neutrophil gelatinase-associated lipocalin
S100A8, S100A9: The S100 calcium-binding proteins, known also as calgranulins A and B or myeloid-related proteins MRP8 and MRP14
MMP-9: Matrix metalloproteinase 9 or gelatinase B, 92 kD
MMP-8: Matrix metalloproteinase 8
TIMPs: Tissue inhibitors of metalloproteinases
Е2: 17*β*-Estradiol
SDS-PAGE: Sodium dodecyl sulfate polyacrylamide gel electrophoresis.
